# The Critical Role and Evaluation of Community Mobilizers in Polio Eradication in Remote Settings in Africa and Asia

**DOI:** 10.9745/GHSP-D-20-00024

**Published:** 2020-09-30

**Authors:** Judy Lewis, Karen LeBan, Roma Solomon, Filimona Bisrat, Samuel Usman, Ahmed Arale

**Affiliations:** a University of Connecticut School of Medicine, Farmington, CT, USA.; b Global health consultant, Washington, DC, USA.; c CORE Group Polio Project India, Gurgaon, India.; d CORE Group Polio Project Ethiopia, Addis Ababa, Ethiopia.; e CORE Group Polio Project Nigeria, Abuja, Nigeria.; f CORE Group Polio Project Horn of Africa, Nairobi, Kenya.

## Abstract

This article assesses the CORE Group Polio Project (CGPP) experience over a 20-year period in 5 countries. It examines how a program designed to provide social mobilization to eradicate one disease, and which did so effectively, functioned within the general framework of community health workers (CHWs). Vertical health programs often have limited impact on broader community health. CGPP has a 20-year history of social mobilization and effective program interventions. This history provided an opportunity to assess how CGPP community mobilizers (CMs) functioned in polio and maternal and child health. The Updated Program Functionality Matrix for Optimizing Community Health Programs tool of the CHW Assessment and Improvement Matrix (AIM) was used to examine CGPP CM roles across different contexts. The analysis determined that CGPP CMs met the basic level of functioning (level 3) for 6 of the 10 components of the AIM tool. This cross-country descriptive analysis of the CGPP demonstrates the importance of embracing the full range of CHW AIM components, even in a vertical program. Use of data, community involvement, local adaptation, and linkage with the health system are especially critical for success. This general lesson could be applied to other community mobilization and disease/epidemic control initiatives, especially as we face the issues of the COVID-19 pandemic.

## Abstract (precis)

Critical community health worker criteria are important for all community programs, including those focused on a single disease. Areas of importance include community engagement, local adaptation, and linkage with the health system—critical areas for current and future epidemics.

## Teaser

**Key Findings:**

- Data use, community engagement, local adaptation, linkage with the health system, and a strong community platform are critical for successful community programming.
- Community-based disease surveillance using local volunteers enhanced national and district efforts.

**Key Implications:**

- Program managers should consider using the Updated Program Functionality Matrix for Optimizing Community Health Programs of the Community Health Worker Assessment and Improvement Matrix (AIM) tool to develop, implement, and assess community health worker programs.
- Policy makers should support and strengthen strong community platforms in disease eradication programs that engage communities, promote local ownership, and use community-level workers.
- Emerging infectious diseases will require community engagement through local mobilizers to implement successful government prevention and response efforts.

## INTRODUCTION

This article examines how a program designed to provide social mobilization to eradicate polio, and which did so effectively, functioned within the general framework of community health workers (CHWs). Although vertical health programs often have limited impact on broader community health, we wanted to assess how well the CORE Group Polio Project (CGPP) community workers functioned in the areas of polio and maternal and child health. We also wanted to examine their roles in different contexts using the components of the Updated Community Health Worker Assessment and Improvement Matrix (CHW AIM).

CGPP has a 20-year history of social mobilization and effective program interventions. Published external evaluations and peer-reviewed articles about CGPP have demonstrated substantial success in increasing oral polio vaccine (OPV) 0 (the newborn dose) and routine OPV and immunization coverage as well as detecting acute flaccid paralysis (AFP) in hard to reach and resistant populations (Table 1).^9^^,^^10^ The experience of CGPP offers many lessons about implementing vertical programs, developing and deploying a cadre of community-level workers, and engaging with the health care system.

**TABLE 1.** Selected Key Indicators of CORE Group Polio Project Program Performance by Country

**Indicator**	**Angola**	**Ethiopia**	**India**	**Kenya**	**Somalia**	**Nigeria**
OPV0 dose	43% card inspection, (2008) to 47% card inspection (2012)^1^	49% (2013) to 59% (2017) compared to regional Ethiopia Demographic and Health Survey data of 15% (2011) to 27% (2016)^2^	36% (2010) to 78% (2017) in Uttar Pradesh within 15 days of birth^3^	64% (2015) to 97% (2017)^4^	95% (2017)^4^	55% (2014) to 99% (2018)^5^
OPV3 among children 12–24 months based on immunization card and mother’s recall	62% (2010)^6^	67% (2012) to 86% (2017) compared with regional state data of 41% (2011) to 50% (2016)^2^	Maintained above 80% coverage in Uttar Pradesh from 2010 to 2017^3^	57% (2015) to 94% (2017)^4^	21% (2017)^4^	47% (2014) to 62% (2017)^4^
Non-polio acute flaccid paralysis rate per 100,000 children under age 15 within 14 days of onset of paralysis with 80% or better stool adequacy	Not available	2.2 (2012) to 2.8 (2017) exceeding national rate of 2.5 (2017)^7^	Not applicable	2.5 (2017)^4^	4 (2017)^4^	13.6 (2014) to 19.6 (2017)^8^

Abbreviation: OPV, oral polio vaccine; OPV0, oral polio vaccine newborn dose; OPV3, oral polio vaccine third dose.

## Situation of Polio at the Inception of CGPP

Mass immunization campaigns in the mid-1990s achieved high levels of polio immunization coverage. However, in some countries, there remained important pockets of children who were repeatedly missed and served as residual pockets of continuing transmission. By 1999, polio geography and incidence had decreased considerably but it was clear that the goal of eradicating polio by 2000 would not be met and that more focused efforts would be needed to address polio “hotspots” (see Losey et al.^11^).

Mass immunization campaigns in the mid-1990s achieved high levels of polio immunization coverage, but some children were repeatedly missed.

Where community involvement was low, OPV coverage remained low. Although millions of temporary volunteers supported mass campaigns, their job ended when the campaign ended. Conflict, political instability, geographic inaccessibility, nomadic and mobile populations, poor infrastructure, and anti-vaccination social and religious beliefs were some of the obstacles that led communities either to refuse immunization or prevent participation, resulting in low routine immunization and OPV rates. Polio experts within United States Agency for International Development (USAID) began to make recommendations for broad social mobilization efforts to increase community participation in the eradication of polio. Experts increasingly recognized that each remaining polio-endemic country offered a unique set of challenges that required local solutions.^12^ CGPP started in 1999 with funding from USAID to address these issues. This process has been well described.^11^

## METHODOLOGY

We used a mixed-methods evaluation approach to compare the evolution of CHWs within CGPP. The 2 first authors collected the data. We began with an extensive literature and document review about CGPP, which included multiple mid- and final evaluations reflecting different grant periods. This review was a major undertaking because of the project’s 20-year history in 11 countries. We also conducted surveys through computer, phone, and in-person interviews with CGPP secretariat directors and staff. The survey included 18 broad questions about program operation, management, and development over time, focusing on community mobilizer (CM) roles. The timeframe for data collection was April 2018 to September 2019. The lead authors were involved in all levels of data collection and analysis. We focused on 5 programs: India, Ethiopia, and Angola, which have been in operation the longest, and Nigeria and Kenya/Somalia (Horn of Africa Program), which are more recent but face particularly challenging situations.

The focus was on 5 programs: India, Ethiopia, and Angola, which have been in operation the longest, and Nigeria and Kenya/Somalia, where conflict and migration were challenges.

We used the Updated Program Functionality Matrix for Optimizing Community Health Programs^13^ of the CHW AIM^14^ as the framework for our analysis. CHW AIM uses 10 programmatic components that have been found to contribute to an effective CHW program. Each of the 10 components is subdivided into 4 levels of functionality: (1) nonfunctional, (2) partially functional, (3) functional, and (4) highly functional. The program also includes a process for creating a participatory functionality score, which we did not use. We used the criteria for level 3 (functional) to examine whether the CM work met the criteria for this level of functioning. This standard was used across country programs and contexts for each of the 10 components to examine the long-term impact of CM roles in each country.

## CGPP COUNTRY PROGRAMS

The 5 CGPP country programs discussed in this article began between 1999 and 2014. Program inception dates and the number of collaborating nongovernmental organizations (NGOs) are provided in Table 2.

**TABLE 2.** CORE Group Polio Project Country Start Dates and Number of Collaborating NGOs, Past and Present^a^

	**Angola**	**Ethiopia**	**India**	**Kenya/Somalia**	**Nigeria**
Year started	1999	2001	1999	2014	2013
Number of international NGOs	6	9	6	5	3
Number of local NGOs	4	10	77	5	8

Abbreviation: NGO, nongovernmental organization.

a The NGOs did fluctuate over time and area covered, so for all data in this article, we have referenced numbers from Losey et al.^11^

### Name and Number of Community Mobilizers

CGPP’s CHWs were CMs, which are sometimes referred to by different names. The term CM is used in this article for all CGPP country programs to distinguish the CGPP cadre from other CHWs used by NGOs and government agencies. Social mobilization was the main strategy to provide polio education, engage communities in polio vaccination, track children missed during OPV campaigns, and conduct AFP surveillance in high-risk populations.

The country-specific names used for the CMs reflected government policy or nomenclature widely used by partners when the program began (Table 3).

**TABLE 3.** Name, Number, and Type of Community Mobilizers by CORE Group Polio Project Country

**Country**	**Name**	**Current Number**	**Type**
Angola	Community volunteers	2,700 (2017 FE)	Part time
Ethiopia	Community volunteers	13,720 (2017 FE)	Part time
India	Community mobilization coordinators	1,100^11^	Part time
Kenya/Somalia	Community health volunteers	1,025 (2017 FE)	Part time
Nigeria	Volunteer community mobilizers	2,200 (2017 FE)	Part time

Abbreviation: FE, Final Evaluation.

As governments developed community health strategies, the project incorporated government CHWs into their programs to help address polio. For example, as the Ethiopia government deployed its Women’s Development Army (WDA), CGPP worked with the volunteer WDA leaders (1 for every 30 WDA volunteers) in CGPP implementation areas where WDA volunteers were active, and the number of CMs greatly increased. Numbers of CMs varied over time as partners, population, or geographic area changed. For example, in Ethiopia, CGPP trained 2,000 CMs between 2004 and 2006; 4,165 between 2007 and 2012; and 13,720 between 2013 and 2017 (this included WDA leaders beginning in 2015).

All projects had part-time CMs who fit the description for CHW-regular described by Hodgins et al.^15^

### Location of Work and Population Reached

In each country, CGPP worked in areas assigned by the in-country Interagency Coordinating Committee (ICC) for Polio Eradication (Table 4).

**TABLE 4.** Location of CORE Group Polio Project Work and Population Reached (Annual Reports and 2017 Final Evaluations)

**Country**	**Location**	**Population Reached**
Angola	5 provinces (Benguela, Bie, Cuanza Sul, Luanda, Malange)	>9 million children under 15
Ethiopia	85 districts in 5 regions (Benshangul-Gumuz; Gambella; Oromiya; Southern Nations, Nationalities and Peoples; Somali) 185 border crossing points^a^	>6 million people of which 1,806,950 are children under age 5^a^
India	58 blocks in 12 high-risk districts of Uttar Pradesh, 2 districts in Assam, and 1 district in Haryana	600,000 households reaching population of 3 million^a^
Kenya/Somalia	Kenya: 7 counties (Lamu, Garissa, Mandera, Marsabit, Turkana, Wajir, and parts of Nairobi) Somalia: 3 border regions (Lower Juba, Gedo, and Bakool)	Kenya: 466,250 children under age 5 Somalia: 109,000 children under age 5
Nigeria	32 local government areas in 5 northern states (Borno, Kaduna, Kano, Katsina, and Yobe) 6 internally displaced persons camps	Approximately 500,000 children under age 5^a^

a Data from secretariat directors.

Areas and population reached with OPV changed during the project, often on short notice, based on reviews of immunization data and/or the need to reach special at-risk and inaccessible populations. Populations were large and often in noncontiguous areas. For example, over 1.4 million people were reached in Nigeria through social mobilization efforts.^4^

Areas and population reached with OPV changed during the project, often on short notice.

## ANALYSIS OF CM FUNCTIONALITY IN THE CHW AIM

The following sections highlight key programmatic components of CM functionality as described in the Updated CHW AIM. Table 5 provides a summary of the matrix and the criteria used to determine whether AIM level 3 functionality was achieved for each component. Similarities and differences between programs are provided below.

**TABLE 5.** Community Health Worker Assessment and Improvement Matrix Tool Components and Criteria Used for CORE Group Polio Project

**CHW AIM 2018: Revised Programmatic Components**	**CHW AIM 2018 Elements** **Examined for CGPP CMs**
1. Role and Recruitment How the community, CHW, and health system design and achieve clarity on the CHW role and from where the CHW is identified and selected. Level 3 requires: Recruitment: CHWs recruited from community and community consulted in selection. Criteria for functionality, attitudes, expertise, and availability of CHWs clearly delineated. Role: Clearly defined and documented, agreed upon by CHW, community, and health system. Workload and location: CHW to population ratio reflects expectations, population density, geographical constraints, and travel requirements.	Recruitment: Initial selection Final decision Type of CMRole: Community mobilization to increase polio and routine vaccination rates Community-based surveillance of acute flaccid paralysis Promote maternal and child healthWorkload and location: Number of work days/week Hours worked Average number of households reached monthly Work locations
2. Training How preservice training is provided to CHWs to prepare for their roles and to ensure they have the necessary skills to provide safe and quality care. How ongoing training is provided to reinforce initial training, teach CHWs new skills, and help ensure quality. Level 3 requires: Initial training: meets global guidelines and occurs within 6 months of recruitment. Continuing education: provided at least annually and vertical topics are integrated	Initial Training: Trainers Content of trainingContinuing education
3. Accreditation How health knowledge and competencies are assessed and certified prior to practicing and recertified at regular intervals while practicing. Level 3 requires: CHW health knowledge and competencies are tested and a minimum standard must be met.	Assessment of CM health knowledge and competencies External program evaluations
4. Equipment and Supplies How the requisite equipment and supplies are made available when needed to deliver expected services. Level 3 requires: Equipment, supplies, and job aids are provided and available for resupply on a regular basis.	Continuous supply of job aids
5. Supervision How supportive supervision is carried out such that regular skill development, problem solving, performance review, and data auditing are provided. Level 3 requires: A dedicated trained supervisor uses checklists to conduct supervision visits at least every 3 months and uses summary statistics to identify areas for improved service delivery.	Type of supervisor Average number CMs supervised Supervisor paid Tools used Frequency of supervision performance evaluation (individual and program)
6. Incentives How a balanced incentive package reflecting job expectations, including financial compensation in the form of a salary and nonfinancial incentives, is provided. Level 3 requires: CHWs are compensated at a competitive rate and receive nonfinancial incentives	Financial (honorarium, transport/food allowance) Nonfinancial (certificates, performance awards, formal recognition, skill development, uniforms, job aids, free access to health services) Community recognition
7. Community Involvement How a community supports the creation and maintenance of the CHW program. Level 3 requires: Community supports, recognizes, and appreciates CHWs. CHWs engage with community structures.	Discuss CM role and selection Provide feedback on performance Solving problems Provide incentives/recognition Ongoing data-based dialogue Use of community influencers Community structure engagement
8. Opportunity for Advancement How CHWs are provided career pathways. Level 3 requires: Advancement is offered to CHWs, training opportunities are provided to learn new skills, and advancement rewards good performance.	Potential for advancement Project, government, community Retention Percentage retained Length of service Reasons for leaving
9. Data How community-level data flow to the health system and back to the community and how they are used for quality improvement. Level 3 requires: CHWs document visits and provide data that are reported to public sector monitoring systems. Supervisors monitor data quality, and CHWs and communities use data in problem solving.	Data collection tools Feedback provided to community and local government Data used for problem solving
10. Linkages to the National Health System The extent to which the Ministry of Health has policies in place that integrate and include CHWs in health system planning and budgeting and provides logistical support to sustain district, regional, and/or national CHW programs. Level 3 requires: Linkages between CHWs and the formal health system (Ministry of Health), including referral, recognition and appropriate CHW provisions.	CM referrals Formal health system recognition and support Country ownership

Abbreviations: AIM, Assessment and Improvement Matrix; CHW, community health worker; CM, community mobilizer.

### Role and Recruitment

#### Recruitment

Table 6 shows that in all countries, CGPP staff provided generic selection criteria to NGOs and/or community leaders, and communities played an important role in identifying candidates. Communities could modify the criteria to best match the local context, such as literacy or sex of the CM. In terms of who chose the CMs, NGOs selected the CMs in Angola since they were already working with them. In Kenya/Somalia, health facility staff also participated in the selection process. In Angola, India, and Kenya/Somalia, the NGO made the final hiring decision. In Ethiopia, community leaders made the decisions with input from health facility staff and district administrators (later by the health extension workers). In Nigeria, ward selection committees decided. In terms of the types of people selected, existing CMs or CHWs were selected when possible in Angola, Kenya/Somalia, and India. In Ethiopia, community leaders and influencers (often religious figures) were selected. Madrasa teachers and elected officials initially helped identify candidates in India to recruit Muslim women.

**TABLE 6.** Recruitment of Community Mobilizers for CGPP Country Programs

	**Angola**	**Ethiopia**	**India**	**Kenya/** **Somalia**	**Nigeria**
Initial selection					
CGPP provides generic criteria	X	X	X	X	X
NGO identifies candidates	X		X		
Community leaders nominate candidates	X	X	X	X	X
Community interviews candidates		X			
Health facility staff				X	
Final decision					
NGO	X		X	X	
Community leaders		X			X
Local government		X			X
Type of CM selected					
Existing CMs	X		X	X	
Community leaders and influencers		X	X		X

Abbreviations: CGPP, CORE Group Polio Project; CM, community mobilizer; NGO, nongovernmental organization.

#### Selection Criteria

Table 7 identifies defining characteristics of CMs. These characteristics varied by cultural context and changed over time. Initially, literacy was prioritized, but later characteristics such as respect and trust by the community and knowledge of local customs and norms were found to be more important by CGPP. NGO staff developed methods for working with CMs with low literacy, such as having their children write in immunization registers or using oral storytelling with the supervisors.

**TABLE 7.** CORE Group Polio Project Country Program Community Mobilizer Selection Criteria: Sex and Literacy^a^

**Country**	**Sex (% Women)**	**Rationale**	**Literacy/Education**
Angola	90%	Women preferred	Low literacy
Ethiopia	89%	Community preference Religious beliefs Insecurity Women’s Development Army Leaders must be women by government policy	55% with basic reading and writing
India	97%	Women preferred	Basic high school education
Kenya/Somalia	29%	Community preference Religious beliefs Insecurity Difficult terrain	Basic reading and writing
Nigeria	99%	Community preference Religious beliefs Insecurity Influence in the community	Some literacy; value of literacy diminished over time

a Data from the Secretariat Directors as of August 2019.

India preferred female community health volunteers since they have easier access to mothers, the principal caregivers for children. In Ethiopia, there were initially more women when the project worked in agrarian districts. When the project moved to border areas and pastoralist and semipastoralist districts, the ratio shifted to equal numbers of women and men. Reasons included religious preference, security issues, women being too busy with household work, and community decision making. The ratio changed again as the government required all WDA volunteers to be women. In Nigeria, project surveys validated that women were seen as the primary caregivers of children, and in conservative Muslim communities, only women could enter another woman’s household. However, the project has realized that because men are often the decision makers about health care, the project should recruit male CMs or more married couples who can work together. Angola also found a need for male CMs for the same reason. In Kenya/Somalia, CM sex varies by setting. In urban areas 60% CMs are women, while in the sparsely populated northern arid counties and borders, only 11% are. The sex of the CM is context based and differs according to nomadic lifestyle, harsh terrain, sparsely populated communities, and community preference.

**Figure:**
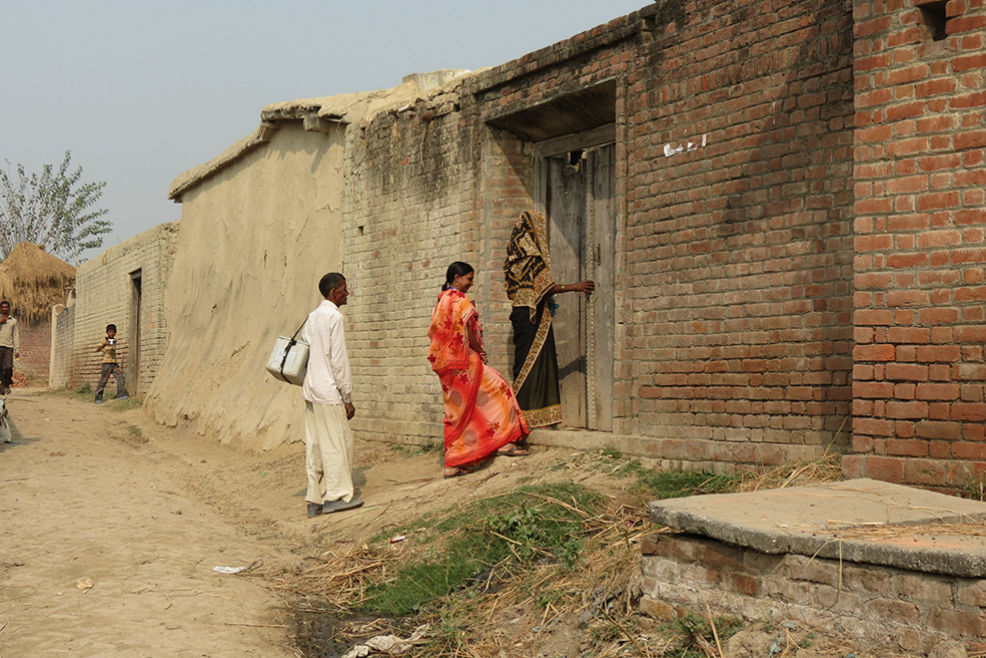
A community mobilizer in India conducting a home visit.Photo credit: © 2012 Rina Dey/CGPP India

Commonalities on basic selection criteria of CMs existed across all projects, including the following:

- Known and respected members of the community
- Willing and committed to the welfare of the community
- Free from bad or corrupt behaviors
- Willing and available to learn and work for little or no money

#### Role

Key CM roles in CGPP included community mobilization to increase polio and routine vaccination rates, community-based surveillance of AFP, data collection, and promotion of maternal and child health. The exceptions were India, which did not include AFP surveillance because the country already had a robust system, and Kenya/Somalia, where CGPP worked directly at health facilities because of staff shortages. All CGPP programs used a wide variety of social mobilization methods to increase polio and routine vaccination rates: household visitation, group counseling, and community activities to dispel rumors and build trust in the health care system. A 2019 evaluation found that CMs engaged community leaders, created relationships with influencers, worked with household caregivers, and changed community attitudes that yielded normative and community change, not just individual behavior change. By identifying and reporting suspected polio cases for later investigation, CMs increased AFP surveillance sensitivity.^16^

Program strategies had to respond in a timely way to specific local challenges and culture. In India, tactics changed as local OPV attitudes shifted from early acceptance, to suspicion and resistance, followed by passive acceptance and growing apathy. The Box provides an example of the evolution of the CM’s role and tasks in India. Edutainment (street theatre and puppet shows) was often used by CMs, but locations shifted over time. In Ethiopia, community trust resulted from content messages addressing traditional beliefs about the spiritual causes of paralysis. In Nigeria, 1,200 community influencers and edutainment such as community clowns were important drivers of behavior change. Kenya/Somalia had CMs meet with community members wherever they gathered, such as at bus stops and schools. Data collection included community mapping and tracking immunization status/defaulters and newborns.

BOX. Evolution of Roles and Tasks of Core Group Polio Program/India Community Mobilizers

Core Group Polio Program (CGPP)/India trained and supported community mobilizers (CMs) to engage and convince communities, especially mothers/caregivers, about the benefits of vaccinating their children repeatedly for polio and to ensure that families were motivated to vaccinate their children for other life-threatening diseases.

Initially, the CM’s primary task was to:

- Mobilize community participation at government-run polio booths—1-day events to immunize all children under-5 at a fixed site on fixed days
- Conduct follow-up visits with families who were missed during a vaccination event

However, in certain remote and underserved areas, community resistance developed to a polio-focused strategy primarily due to frustration with and distrust of government, religious fatwas by Muslim leaders, and frustration at the lack of health services resulting in high numbers of sick children.

To overcome the resistance, CMs had to broaden their role:

- Conduct monthly house-to-house visits often over years
- Facilitate community group meetings (such as mothers’ groups, religious groups)
- Use key community sites such as mosques, schools, and festivals for polio-related education, counseling, and problem solving
- Develop detailed maps of their communities and identify houses with unvaccinated children and later newborns
- Maintain immunization status records for all under-5 children in their areas
- Recruit community, cultural, and religious leaders to accompany them during household visits and to act as credible communication sources to dispel fears and rumors

As CMs learned more about community concerns, CGPP built CM capacity in counseling and in how to use community-relevant training materials and job aids.

The CM’s role grew to include:

- Promoting a larger package of services that responded to community needs and underlying causes of polio transmission, including routine immunization, water and sanitation, control of diarrheal disease, and breastfeeding.
- Referring and accompanying families to health facilities, building trust in the health system.
- Reaching migratory communities that had limited access to information or health services and were at risk of spreading the virus

∘Identifying and training key informers, such as barbers, employers, shopkeepers, and others, who knew the location and movement of migrant families in their area.
∘Developing maps of these populations and vaccine-eligible children, updating the maps regularly with socioeconomic information, and making regular visits to the mobile sites.
∘Forwarding information to government immunization teams to come to the sites to vaccinate the children.

Source: Dey R, Mahendra VS, Morry C, et al. *Influencing Change: Documentation of CORE Group’s Engagement in India’s Polio Eradication Programme.* India: CORE Group; 2018.

**Figure:**
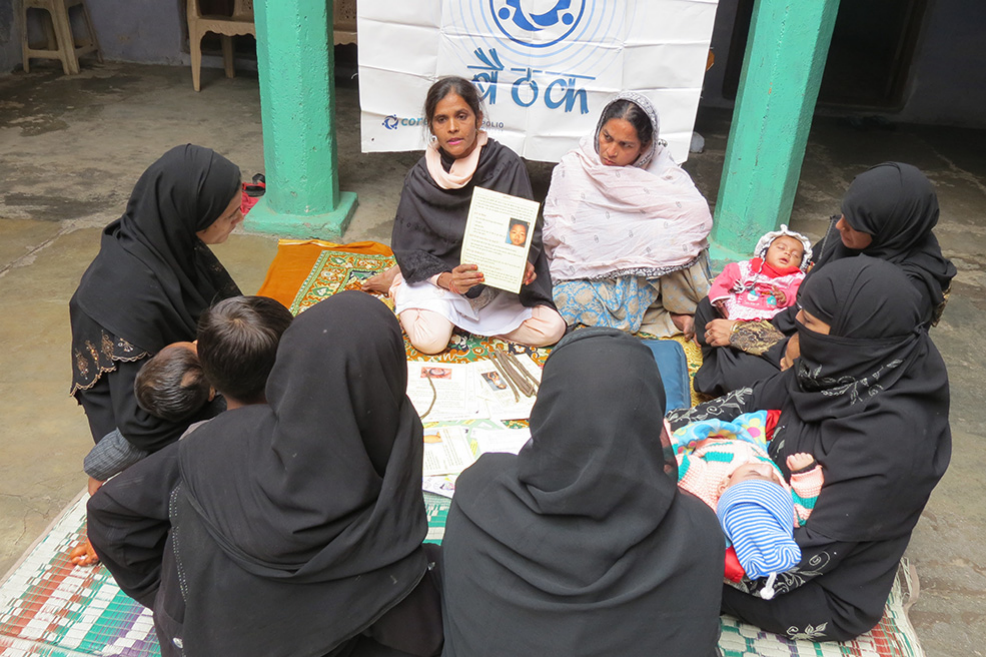
Community mobilizers in India educating mothers on polio vaccination.Photo credit: © 2012 Rina Dey/CGPP India

Although Angola added polio activities to existing duties of child survival CHWs, other countries initially focused solely on polio eradication. How-ever, over time, communities complained that other pressing health needs were not being met, so CGPP began to address other maternal and child health issues, as well as water and sanitation through their CMs.

#### Workload

Table 8 shows that workload varied by country and within country depending on terrain, culture, population density, and community traditions. In Angola, Ethiopia, Kenya/Somalia, and Nigeria, work averaged between 2 and 4 days per week and from 2 to 5 hours per day worked. However, CMs were expected to be available full-time during polio campaigns and other special events. Median average hours per month for CMs ranged from 16 in Ethiopia to 80 in India, and median average households reached monthly ranged from 75 in Angola and Ethiopia to 450 in India. As shown in Table 8, median average for hours per month was directly related to the number of households for which a CM was responsible. Variations in coverage included pastoralist areas in Ethiopia, shared workloads in Nigeria, and changes in program strategies over time. In India, initial work was only 1 week/month during campaigns, but after 2003, the workload increased with social mobilization.

**TABLE 8.** CORE Group Polio Project Community Mobilizer Workload, by Country Program

**Days of Work and Household Coverage**	**Angola**	**Ethiopia**	**India**	**Kenya/Somalia**	**Nigeria**
Average days per week	2–3	2	5	3	4
Average hours per day/worked	2–4	2	4	2–4	4–5
Median average hours/month	30	16	80	36	72
Median average households/month	75	75	450	100	225
Range of households reached monthly	50–100	50–100	400–500	100	150–300

Broad social engagement required working with multiple sectors of the community, and each country developed new strategies. Angola, Ethiopia, India, and Nigeria worked with faith leaders and faith communities. Nigeria worked with traditional leaders. India worked with teachers and children in schools, barbers, mothers’ groups, community influencers, and brick kiln owners. Ethiopia facilitated traditional coffee ceremonies and met with people at encampments, food aid sites, and markets. Nigeria conducted activities in the village square, motor parks, and markets, as well as public holiday gatherings.

Broad social engagement required working with multiple sectors of the community, and each country developed new strategies.

### Training

Training varied by country and situation, but all CGPP programs conducted initial training immediately after CMs were recruited. Angola had the longest training period (2 weeks) to prepare CMs as both child survival and polio workers. Ethiopia, India, Kenya/Somalia, and Nigeria had initial trainings from 3 to 5 days, which was sometimes residential. Training initially focused on polio and immunization, community/social mobilization and interpersonal communication, but in each country the content expanded to additional maternal and child health topics. Although CGPP and NGO staff were the primary trainers, the curriculum was developed with government, UNICEF, and WHO staff as well as experts from health facilities, local governments, and/or international agencies.

Trainings evolved from lectures and presentation into very participative and creative sessions with role playing and household visits, flipbook messaging, dealing with body language, arguments, showing respect, and active listening. Continuous training occurred in all countries through supportive supervision, monthly CM meetings in India, Ethiopia, and Nigeria, and annual CM meetings in India and Kenya/Somalia. The first final evaluation^17^ recommended that CGPP update and strengthen its CM curriculum, and expand CM capacity in Ethiopia and India, by increasing the frequency of refresher training.

### Accreditation

In each CGPP program, the supervisor periodically assessed health knowledge and competencies but no certification system was in place. However, each country program had an outside Know-ledge, Practice, and Coverage evaluation of the CMs, verified by community recollection of receiving CM messages and support. For example, in India in 2011, 97% of mothers with a child 12–23 months knew their CM, and homes visited by CMs had higher levels of routine immunization than children whose homes were not visited.

### Equipment and Supplies

All CGPP countries provided a continuous supply of job aids, which included flip books, registers, writing books, pens, posters, and sometimes bicycles.

### Supervision

In each country, CGPP had a supervision system that reached upward to national and/or state government oversight. CGPP and project staff trained supervisors with expert and government input. The only variability between countries was the number of CMs supervised. This number ranged from 3–5 in Ethiopia to 12–15 in India. All project supervisors were paid or received a stipend. CGPP hired supervisors in all countries except for Ethiopia. In Ethiopia, the health extension workers (HEWs) are trained and supervised by the government; each HEW supervised CMs and 20–35 volunteer WDA leaders. India developed a 4-day master training course for supervisors. All projects used supervisory checklists and several types of data registers (immunization status records, pregnancy tracking, households visited).

The first final evaluation for 1999–2007^17^ recommended more supportive supervision to address CM performance gaps. By 2017, supervision across projects was consistent, with supervisory checklists, registers, on-the-job visits, monthly and quarterly meetings, and performance evaluations in all countries.

Supervision tools, forms, templates, and training manuals can be found at the CORE Group website: https://coregroup.org/polio-eradication-toolkit/.

None of the CMs in any country received a regular salary. However, India, Nigeria, and Kenya/Somalia (except for urban areas) provided a monthly honorarium (average $30–$35), which enhanced motivation but sometimes created discontent when other projects provided a higher monthly amount. The Indian CGPP secretariat helped its mostly female cadre open bank ac-counts and deposited the funding electronically to ensure that the women had more control over their money. Angola, Ethiopia, Kenya/Somalia, and Nigeria provided a daily transport and food allowance (cash or food) during campaign days or CM meetings. In Angola, this allowance was paid by the government.

A mix of nonfinancial incentives evolved over time in all countries. These were provided at 3 levels: country program, government, and community. Each country program was encouraged to introduce new incentives to boost morale and celebrate CM achievements. All provided CM training and skill development, which was highly motivational. All programs provided branded uniforms to identify and motivate the CM. Items included polio branded t-shirts, gowns, aprons, wrap-around skirts, umbrellas, rubber boots, coats, bags, streamers, caps, and scarves. In Nigeria, the CMs were given pink hijabs and they became known as the “pink ladies.” Certificates of recognition were given to CMs in Ethiopia, India, and Kenya/Somalia. Government recognition included officials providing CMs with formal recognition at public events in India and Nigeria. Ethiopia, India, and Nigeria gave performance-based awards to CMs, including mobile phones, radios, and shoes in Ethiopia; plaques in Nigeria; and trophies in India. Kenya/Somalia provided free access to health services for CMs and their dependents.

A mix of nonfinancial incentives evolved over time in all countries to boost morale and celebrate CM achievements.

Community recognition was especially important to CMs and program staff in all the countries. It was considered a major motivator and essential to the work of CMs. In Kenya/Somalia, CMs were seen as community resource people and were invited to participate on committees. In Nigeria, some parents named their babies after the CMs. In India, CMs were recognized at community jamborees, where CMs received gratitude from the community for their referrals and other services to families. Ethiopia CMs were recognized by providing a certificate for their participation and contribution as volunteers.

### Community Involvement

As previously described, the community was involved in every aspect of supporting the CM including recruitment and selection; feedback; problem solving, especially related to resistant households; incentives and recognition and ongoing dialogue about polio data.

The community was involved in every aspect of supporting the CM.

Four of the programs (all but Angola) had a deliberate strategy of identifying and training community influencers to support the CMs. The community influencers helped solve problems with resistant households and monitored the effectiveness of the CM. Mothers’ groups met to discuss their polio indicators compared with other areas. Children were mobilized to be campaign advocates.

Ethiopia worked with religious and clan leaders, traditional healers, and ward (kebele) leaders to counter rumors. In Nigeria, as the project evolved, men were trained as peer informants for polio education and advocacy. In all countries, communities extended appreciation and respect for the CMs.

The content discussed in trainings and at supervision events emphasized concerns of the local community.

### Opportunity for Advancement

Little potential existed for CM advancement within projects because the programs moved frequently to contain the virus. However, exceptional CMs had an opportunity to advance to the next level in India and Nigeria. CMs could become part of the CHW government cadre in India, Ethiopia, and Kenya. In many countries, because CMs were respected, they were invited to other community positions.

In many countries, because CMs were respected, they were invited to other community positions.

Ethiopia, India, and Nigeria all had very high retention rates (86%–95%) in difficult areas (there was no increase in compensation for this work). In Nigeria, experienced CMs delivered more CM messages during household visits than less experienced CMs.^18^ In Kenya/Somalia, retention was only 40% due to the nature of pastoralist communities crossing borders to follow herds and men finding paying jobs.

### Data

CGPP commissioned 3 external final evaluations (1999–2007, 2007–2012, 2012–2017), each of which had recommendations for improving program performance. Initially, CGPP reported on achievements using primarily quantitative counts of activities with supporting anecdotes. In 2008, financial support was provided for baseline household surveys in local service areas and each country added a monitoring and evaluation officer.

In all countries, CMs maintained community maps, registers of pregnant women and newborns, defaulters, child immunization status, and households visited. These maps and registers were shared with health facility staff and next levels of government during supervision visits. Feedback was provided at community meetings and during local government meetings in countries such as Ethiopia and Nigeria. In India, data were posted on a community board. Copies of registers for each country can be found at https://coregroup.org/polio-eradication-toolkit/. In addition, CGPP carried out household surveys in all countries to verify information. In India, CGPP used a census-based management information system for collecting prospective and retrospective information for planning, monitoring, and evaluation of its social and behavior change communication activities. India also used Lot Quality Assurance Sampling surveys and Barrier Analysis to identify impediments to adopting healthy behaviors.^3^

### Linkages to the Health System

#### CM Referrals

All countries had a referral system for polio, and most programs evolved over time to provide referrals for routine immunization, antenatal care, newborns, and childhood and adult illnesses. In all countries, referral was viewed as one of the most important CM activities and one that led to community recognition. CMs were guides for vaccination teams to household defaulters. In many countries, CMs accompanied clients to the nearest health facility. In Kenya/Somalia, CMs guided pastoralists to the nearest health facility once they crossed the border, and they reported animal health issues to health facilities and veterinarians. In Angola, CMs gave caregivers referral slips for the health facility that could then be tracked, allowing the project to assess client follow-through.

All CGPP programs had a referral system for polio, and most systems evolved over time to provide referrals for other health concerns.

#### Formal Health System Recognition and Support

CGPP programs worked to connect with health facilities, health workers, and government agencies in all countries. When CGPP started in Angola, the project actually supported the health system because of conflict and limited government functionality. When the India program started, the government’s community health approach did not include accredited social activists (ASHAs). Auxiliary nurse midwives (ANMs) were the vaccinators, and the CMs guided them to resistant households during supplementary immunization activities. Over time, the program established coordinating meetings under the ANM with anganwadi workers (focused on food supplements and nutrition), ASHAs, and CMs.

When CGPP began in Ethiopia, NGOs were working with CHWs for child survival projects, although all were using different approaches. In 2003, the government began its HEW program training full-time salaried women for 1 year. In 2005, 35,000 HEWs were deployed to work, with 2 HEWs stationed at each health post serving about 5,000 people. CMs became HEW guides in the community, and they planned together at the health facilities. In 2012, the government introduced the volunteer WDA to promote health, education, and agriculture. Some CMs became WDA leaders. In areas without WDA Leaders, CGPP continues to work with its CMs. CGPP found that WDA volunteers, given their other tasks, were not as effective at polio work as polio-focused CMs.

CGPP in Nigeria coordinates with the government structure at the federal, state, and LGA local (ward) levels. The program responds to calls from the government for additional support. For example, CGPP participated in a massive 8-day inactivated polio vaccine campaign in 2014 in the conflict-affected zones of Borno and Yobe states, the largest polio campaign in a conflict area in Africa, reaching nearly 800,000 children.^19^

In Kenya/Somalia, CGPP attached itself to health border facilities and established cross-border health committees primarily to address polio but also to address outbreaks of cholera and other diseases. CGPP provides transportation to health facility staff for immunization outreach targeting high-risk mobile populations along the border. In Kenya, community health extension agents, community health assistants, and health facility staff help monitor the CMs.

When new government cadres were introduced, some tension with CMs arose, but it was reduced with collaboration and communication over time.

When CGPP started, donors and governments thought polio would be quickly eradicated, so health ministries did not anticipate long-term investment in polio CMs. In countries that ended polio work, such as Angola, CMs were not absorbed into a government system. However, after 20 years of CGPP polio work in these countries, country governments are developing plans to integrate some of the CMs into their strategic health plans.

When new government CHW cadres were introduced, some tension with CMs arose, but it was reduced with collaboration and communication over time.

## DISCUSSION

CGPP has worked with populations that were often resistant to immunization and required multiple doses of OPV. These populations were in difficult-to-access places due to conflict, rough terrain, and lack of roads. In addition, health services, staff, and infrastructure were limited, and people spoke multiple languages within countries and followed different tribal customs. Eventually in some areas, CGPP found it necessary to establish cross-border programs with multiple immunization sites at various border-crossing points.

Although many countries hired part-time community workers to assist in polio campaigns, all CGPP countries built the capacity of community residents to volunteer part time all year long and to make house-to-house visits. The CGPP CM strategy, based on CHW work in child survival programs, evolved over time. Initially, Angola added polio interventions onto existing CHW tasks, Ethiopia focused primarily on surveillance for AFP, and India focused on social mobilization. Learning took place from technical assistance visits from CGPP headquarters and secretariat staff, and later from a set of midterm and final evaluations, conferences and workshops, and a growing literature on CM best practices. Learning was eventually codified into templates, curricula, and articles that benefitted more recent projects in Nigeria and Kenya/Somalia. Each country adap-ted its CM practices to respond to local needs. This adaptation reflects similar findings that the CM evidence base needs to be contextualized for different places and situations.^20^

All AIM components, with the exception of accreditation, were used. Three were partially met and 6 achieved basic functionality (Table 9). Of the set of 10 components, data, both quantitative and qualitative were critical to program performance. In 2008, CGPP developed community-based health information systems and added a monitoring and evaluation officer to each country. Indicators moved from counting activities, houses, and people to providing useful household information aggregated at community and district levels around key global polio and immunization indicators that improved decision making. A focus on supervision systems, robust data collection systems combined with periodic surveys, and performance assessments created functioning CM systems over time. Strategic use of data at every level, from planning and message development to results monitoring, was previously highlighted as one of the 4 lessons learned from the CGPP/UNICEF India experience.^21^

**TABLE 9.** CGPP Achievement of Community Health Worker Assessment and Improvement Matrix Tool Components

**CHW AIM 2018: Revised Programmatic Components**	**CGPP Achievement of Level 3 Functionality**
1. Role and Recruitment How the community, CHW, and health system design and achieve clarity on the CHW role and from where the CHW is identified and selected.	Level 3 achieved Clarity and clear criteria identified for recruitment and role. Some criteria changed over time.
2. Training How preservice training is provided to CHWs to prepare for their roles and ensure they have the necessary skills to provide safe and quality care. How ongoing training is provided to reinforce initial training, teach CHWs new skills, and help ensure quality.	Level 3 achieved Initial training in 4 of 5 programs 3–5 days, maximum was 2 weeks in Angola. Trainers included CGPP and NGO staff with health facility and government officials and other resource people varying. Training content in addition to polio, provided broad maternal and child health and social and behavior change skills in most programs. On-the-job mentoring was the major method of continuing education through CM mentoring, monthly meetings, and annual meetings.
3. Accreditation How health knowledge and competencies are assessed and certified prior to practicing and recertified at regular intervals while practicing.	Level 3 not achieved because there was no formal certification system. CM health knowledge and competencies assessed initially and periodically. External program evaluations documented Knowledge, Practice, and Coverage of CMs and verified with community.
4. Equipment and Supplies How the requisite equipment and supplies are made available when needed to deliver expected services.	Level 3 achieved Continuous supply of job aids (flip books, registers, writing books, pens, posters, sometimes bicycles).
5. Supervision How supportive supervision is carried out such that regular skill development, problem solving, performance review, and data auditing are provided.	Level 3 achieved All country programs addressed supervision at all levels and types.
6. Incentives How a balanced incentive package reflecting job expectations, including financial compensation in the form of a salary and nonfinancial incentives, is provided.	Level 3 partially achieved CMs were part-time workers and did not receive a salary. In 3 of 5 programs, CMs received a monthly honorarium (underpaid compared to UNICEF). All provided transport/food allowances for campaigns and program meetings. 3 of 5 provided certificates and performance awards. 1 provided free access to health services. All had community recognition.
7. Community Involvement How a community supports the creation and maintenance of the CHW program.	Level 3 achieved This was one of the strongest components of the CGPP. All programs demonstrated strong and continuous community involvement.
8. Opportunity for Advancement How CHWs are provided career pathways.	Level 3 partially achieved Because the program was vertical and had changing geographic areas, opportunities to advance within the program were limited. 3 of 5 programs reported opportunities in government and community. Retention was high (86%–95%) in 3 of the programs, 40% in another, no data in the fifth.
9. Data How community-level data flow to the health system and back to the community, and how they are used for quality improvement.	Level 3 achieved Data collection tools included community maps, registers of pregnant women and newborns, defaulters, child immunization status, and household visits. Feedback was provided to community and local government and health system. Data were used for problem solving to improve program performance.
10. Linkages to the National Health System The extent to which the Ministry of Health has policies in place that integrate and include CHWs in health system planning and budgeting and provides logistical support to sustain district, regional, and/or national CHW programs.	Level 3 partially achieved Because program was vertical and had limited time expectations, it was never fully integrated with the national health system even though CM referrals were made and CMs worked closely with government cadres in all countries.

Abbreviations: AIM, Assessment and Improvement Matrix; CGPP, CORE Group Polio Project; CHW, community health worker; CM, community mobilizer; NGO, nongovernmental organization.

CGPP Nigeria rapidly improved its OPV 0 dose by asking its CMs to attend “naming ceremonies” since so many women gave birth at home. CGPP Ethiopia increased its OPV 3 coverage to double that of non-CGPP areas within the same state by focusing on messaging to lower the drop-out rate.^2^ CGPP India increased its full routine coverage in CGPP catchment areas of Uttar Pradesh from 48% (2008) to 78% (2017), well above Uttar Pradesh’s state coverage (51% in 2016) by using local data from CGPP’s census-based management information system.^3^ India conducted barrier analysis to examine factors responsible for timely OPV 3, finding that respondents who perceived other benefits of child immunization were 3 times more likely to timely vaccinate their children than those who did not, allowing them to adapt their messaging.^22^ Each country commissioned Knowledge, Attitude, and Practice surveys of the CMs and community residents that provided data for new messaging, refresher training, and increased supervision. High-quality information and data analysis increased credibility of CGPP with the formal health system over time. The creation and implementation of high-quality data collection, analysis, and the utilization of the information for program improvement was a major contribution of CGPP. These steps are essential for ongoing routine immunization efforts in all countries.

Community involvement was the bedrock of the program and the original impetus for having CGPP work in remote settings. Communities were integrated throughout several AIM components in which functionality was met. Other research has found that CHWs are embedded in the community when community members trust and respect them and feel a sense of ownership over the program.^1^ CMs were from the same community and ethnicity, identified by the community, and community members contributed ideas and supported CMs in their tasks. CMs did not work in isolation; in all cases examined, they required the support of community leaders and influencers. This need for support was especially true in areas where trust in government health programs was low or families were vaccine-hesitant. The CGPP strategy depended on CMs providing behavioral change visits to households most at risk of missed vaccination, using detailed community maps. Evidence supports the conclusion that these house-to-house visits played a role in increasing OPV 3 completion in Ethiopia and India.^6^ In 2013, the Global Polio Eradication Initiative made a new strategic plan, which recognized that the conventional eradication strategy needed to be supplemented by efforts to increase community participation according to local needs for a multipronged, area-specific strategy that would vary in different settings.^23^

Community involvement was the bedrock of the program and the original impetus for having CGPP work in remote settings.

Communities participated in the CM role definition and recruitment, training, appropriateness of equipment and supplies, and incentives (community recognition was a major support). The sex of the CM was dependent on the community’s religious and cultural preferences. Women were often selected because of their role in family caregiving or access to households, especially in Muslim communities. Men were selected because of geographic and population challenges such as harsh terrain, pastoral movement, limited phone networks, or conflict and insecurity. All programs recognized the need to include both men and women for social mobilization and decision making to improve vaccination and other child health indicators, and they developed community influencer strategies to support CM work. The roles of CMs expanded over time to include perceived needs of the community (e.g., water, sanitation, roads, antenatal care, newborn care, injuries). Validation of community-defined issues and responsiveness to them improved vaccination rates and demonstrated the need for integrated services in vertical programs, similar to previous findings.^20^ In countries where community-based surveillance of AFP was prominent, non-polio AFP rates expanded in CGPP communities, often exceeding the national rate (see Table 1). In addition, the community-based AFP system allowed for rapid identification and response to other disease outbreaks such as chikungunya and measles.

The roles of CMs expanded over time to include perceived needs of the community (e.g., water, sanitation, roads, antenatal care, newborn care, injuries).

Linkages to the health system component were only partially functional. Because CGPP was a vertical program with limited time expectations, it was never fully integrated into the national health system. However, the support of government and the ministry of health at the national and state level and the strong linkage of the CM program to local government leaders and committees were essential to polio eradication in these hard-to-reach communities. Referrals to health facilities for immunization and other illnesses were viewed by CMs as one of their most important duties, which was an incentive. The partnership worked both ways. Influential government leaders reduced some of the rumors and hesitancy and provided the CMs with recognition. CGPP trained government health workers to supervise and utilize the skills and local knowledge of the CMs. Ludwick et al.^24^ noted that factors pertaining to supportive supervision and relationships with other health care workers related to variances in performance outcomes within a program.

CGPP’s country programs demonstrate how a CHW model can be utilized in a vertical program and adapted to meet specific country and community needs. Other research identified 4 essential elements for an enabling CHW work environment that were also found in this project: workload, supportive supervision, supplies and equipment, and respect from the community and the health system.^25^ The CHW AIM Tool proved useful in systematically assessing CHW functionality of a vertical program. Others have found it useful as a participative exercise with village health teams in Uganda for integrated programs.^26^

## CONCLUSION

This cross-country analysis of the CORE Group Polio Program’s Community Mobilizers demonstrates the importance of the full range of AIM components, even in a vertical program. It also suggests that vertical programs need to expand to address community needs if they are to be effective in meeting their original goal. Data, including local registration of vital events and child registries, played a critical role in program improvement and constitute an essential component. Community engagement is also critical to address misinformation, vaccine hesitancy, and mistrust of government—such engagement needs to be tailored to each culture and community. Community-based surveillance using local volunteers, especially in hard-to-reach populations, enhanced national and state efforts. Partnerships and communication with government health systems are important for program credibility, success, and sustainability.

These lessons are important at this point in time because of the variety of vertical programs and disease challenges from measles, Ebola, and the current COVID-19 pandemic, as well as noncommunicable diseases. There is value in using a similar approach to that used by CGPP and its CMs for responding to COVID-19, as well as other global public health priorities.^27^ Responses to COVID-19 should engage the community through its community mobilizers for nuanced and repeated messaging and discussion to improve the knowledge and attitudes of different community groups about the virus and to keep their trust. The CM’s role can add value to government efforts on disease prevention, testing, contract tracing, home visiting, and community support. Once a vaccine is developed, CMs could mobilize communities for high vaccination coverage.

## Notes

**Peer Reviewed**

**First published online:** August 3, 2020
